# Detection of *Mycobacterium kansasii* using a combination of loop-mediated isothermal amplification (LAMP) and lateral flow biosensors

**DOI:** 10.1007/s10123-020-00143-z

**Published:** 2020-09-02

**Authors:** Chuang Chen, Jia Lu, Bo Long, Zhengyuan Rao, Yuan Gao, Weina Wang, Wenfeng Gao, Jun Yang, Shu Zhang

**Affiliations:** Sichuan Center for Disease Control and Prevention, Chengdu, 610041 Sichuan Province China

**Keywords:** *Mycobacterium kansasii* (*M. kansasii*), Loop-mediated isothermal amplification (LAMP), Lateral flow biosensor (LFB)

## Abstract

*Mycobacterium kansasii* is an opportunistic pathogen that causes both intrapulmonary and extrapulmonary infections. The symptoms of the pulmonary diseases caused by *M. kansasii* closely resemble *Mycobacterium tuberculosis*. Rapid and accurate differentiation of *M. kansasii* from *M. tuberculosis*, as well as other mycobacteria, is crucial for developing effective therapeutics and disease treatment. In this study, we combined loop-mediated isothermal amplification (LAMP) with lateral flow biosensors (LFB) to detect *M. kansasii*, by targeting the species-specific sequence of *rpoB*, a gene which encodes the β subunit of bacterial RNA polymerase. The assay was validated to ensure that it was highly selective by testing *M. kansasii*, *M. tuberculosis*, other species of respiratory bacteria, and other nontuberculous mycobacteria. The detection limit of the assay was 1 fg/μL of DNA and 50 CFU of bacilli in sputum. The *M. kansasii*-LAMP-LFB assay is a fast, cheap, and accurate method for detecting *M. kansasii* by constant temperature amplification and simple interpretation.

**Electronic supplementary material:**

The online version of this article (10.1007/s10123-020-00143-z) contains supplementary material, which is available to authorized users.

## Introduction

Nontuberculous mycobacteria (NTM) comprise most mycobacteria except the *Mycobacterium tuberculosis complex* (MTBC) and *Mycobacterium leprae*. With an increase in the number of patients suffering from immune injury (HIV infection, organ transplant patients, etc.) (Griffith et al. 2007), the number of NTM diseases has increased significantly (Wassilew et al. 2016). NTM are widely distributed in the environment, mainly in soil and water, and are classified into fast-growing and slow-growing mycobacteria. *Mycobacterium kansasii*, an opportunistic pathogen, is a slow-growing NTM. Patients with chronic lung disease, mycobacterial disease, malignancy, and alcoholism have a higher risk of developing diseases caused by *M. kansasii* (Kim et al. 2019; Hirashima et al. 2014; Theodore and Charles 2002; Won-Jung et al. 2002). In China, *M. kansasii* is the third most common slow-growing NTM to cause clinical disease (Fang et al. 2019; Tan et al. 2018).

As one of the most important pathogenic nontuberculous mycobacteria, the clinical symptoms of *M. kansasii* infection are similar to those seen in patients with tuberculosis and other nontuberculous mycobacterial infections, but the treatment is different. It is therefore very important to avoid misdiagnosis of *M. kansasii* and to differentiate it from other mycobacteria. The traditional method of identifying *M. kansasii* is through biochemical identification after culturing, which is time intensive and yields in accurate results. Chromatography, including gas chromatography, high-pressure liquid chromatography, and time-of-flight mass spectrometry that have been used to detect *M. kansasii*, require expensive equipment and complex operation (George et al. 2018; Lau et al. 2015; Olivier and Loots 2012). Similarly, the molecular methods, such as GeneChip, reverse hybridization, and sequencing technology that have been used to identify *M. kansasii*, are still relatively expensive because of the costly instruments required, time consumption, and complicated procedures (Quezel-Guerraz et al. 2010; Suhail and Eiman 2019; Seagar et al. 2008).

The loop-mediated isothermal amplification (LAMP) method combined with use of a lateral flow biosensor (LFB) is a method of multiple cross-constant temperature amplification, which uses the LFB to observe the results using the naked eye (Wang et al. 2017; Wang et al. 2014). This method can be directly applied for the detection of clinical samples. According to previous studies, *M. kansasii* can be distinguished from other mycobacteria by targeting species-specific sequences, such as 16SrRNA, heat shock protein 65 (*hsp65*), RNA polymerase beta subunit (*rpoB*), and 16S-23S rRNA gene internal transcribed spacer (ITS) region (Chikamatsu et al. 2019; Rina et al. 2014; Zoe et al. 2011). In the current study, we developed a LAMP-based method to detect *M. kansasii* by targeting *rpoB*.

## Materials and methods

### Visual detection of LAMP products using the lateral flow biosensor

A total of 0.5 μL of LAMP products and 100 μL of running buffer (10 mM PBS, pH 7.4 with 1% Tween-20) were deposited, separately, in the designated region of the lateral flow biosensor (Hai Tai Zheng Yuan Technology Co., Ltd., Beijing, China). Once the biosensor had absorbed the entire quantity of running buffer (~ 2 min), the subsequent detection of the LAMP products was computed in the form of red lines on the nitrocellulose filter membrane (NC membrane) of the lateral flow biosensor (LFB). The TL was test line, and the CL was control line. The principle of LAMP product detected in LFB was reported (Wang et al. 2016); on the NC membrane of LFB, two zones as the test zone (conjugated with anti-FAM) and control zone (conjugated with biotin-BSA) would be combined with FAM and biotin in the amplified product to visualize the result in the form of red lines on the NC membrane.

### Preparation of strains and genomic template

The 85 mycobacteria and 3 respiratory pathogenic bacteria used in this study (Table 1) were obtained from the National Tuberculosis Reference Laboratory (NTRL) of China. All mycobacteria were cultured at 37 °C on modified Loewenstein-Jensen medium, purchased from Celnovte-Bio Co. Ltd. (Zhenzhou, China). Genomic DNA from these strains was extracted using the CTAB-phenol-chloroform extraction method. The CTAB was purchased from Amresco (USA). The genomic DNA was quantified using a Nanodrop ND-100 instrument (Calibre, Beijing, China) and diluted serially to 1 ng/μL, 10 pg/μL, 1 pg/μL, 100 fg/μL, 10 fg/μL, 1 fg/μL, and 0.1 fg/μL to analyze the detection limit of the *M. kansasii*-LAMP-LFB assay.

**Table 1:** The mycobacteria used in this study

S. no	Bacteria	Source of strain	Number of strains
1	*M. kansasii*	ATCC 12478	1
		Isolated strains (NTRL)	24
2	*M. tuberculosis*	H37Rv (ATCC 27294)	1
		Isolated strains (NTRL)	11
3	*M. intracellulare*	ATCC13950	1
		Isolated strains (NTRL)	3
4	*M. chelonae*	ATCC14472	1
		Isolated strains (NTRL)	2
5	*M. fortuitum*	ATCC6841	1
		Isolated strains (NTRL)	2
6	*M. gordonae*	ATCC14470	1
		Isolated strains (NTRL)	2
7	*M. abscessus*	ATCC19977	1
		Isolated strains (NTRL)	5
8	*M. aurum*	ATCC23366	1
9	*M. neoaurum*	ATCC25795	1
10	*M. marinum*	ATCC927	1
11	*M. gilvum*	ATCC43909	1
12	*M. aichiense*	ATCC27280	1
13	*M. smegmatis*	ATCC19420	1
14	*M. para fortuitum*	ATCC19686	1
15	*M. terrae*	ATCC15755	1
16	*M. nonchromogenicum*	ATCC19530	1
17	*M. vaccae*	ATCC15483	1
18	*M. avium*	ATCC25291	1
		Isolated strains (NTRL)	4
19	*M. phlei*	ATCC11758	1
20	*M. scrofulaceum*	ATCC19981	1
21	*M. gastri*	ATCC15754	1
22	*M. triviale*	ATCC23292	1
23	*M. xenopi*	ATCC19250	1
24	*M. africanum*	ATCC25420	1
25	*M. bovis BCG*	ATCC 19274	1
26	*M. tuberculosis*	H37Ra (ATCC 25177)	1
27	*M. bovis*	ATCC19210	1
28	*M. malmoense*	ATCC29571	1
29	*M. arupense*	Isolated strains (NTRL)	1
30	*M. kumamotonense*	Isolated strains (NTRL)	1
31	*M. paragordonae*	Isolated strains (NTRL)	1
32	*M. scrofulaceum*	Isolated strains (NTRL)	1
33	*K. pneumoniae*	Isolated strains (NTRL)	1
34	*N. meningitidis*	Isolated strains (NTRL)	1
35	*S. pneumoniae*	Isolated strains (NTRL)	1

### Design of *M. kansasii*-LAMP-LFB assay primers

The set of primers used for the *M. kansasii-*LAMP-LFB assay were designed by Primer ExplorerV4 (Eiken Chemical, Japan) and the primer software PRIMER PREMIER 5.0, based on the *rpoB* reference sequence (GenBank accession no. NC_022663.1). The primer sequences shown in Table 2 were further analyzed by BLAST and confirmed to be specific for *M. kansasii*. The locations of the primers are shown in Figure S1.

**Table 2:** The set of primers used for the *M. kansasii-*LAMP-LFB assay in this study

Primers	Sequences (5′ primer)	Length
F3	GGCAATGTCGATGACAACAG	20
B3	ACATCGGCCAGATCCTG	17
FIP*	CCGAGCCGAACCAGATCGTGCTGCAGTTCGGCCTCCT	37
BIP	AAGGTTGGCCGCCCAGTAAACCCACCTGGGATGG	34
LF*	GACTCCGGTGTTCGA	15
LB	GTGAACCCGCGATCT	15

FIP*, 5′-labeled with 5-carboxyfluorescein (5-FAM) used in LAMP-LFB assay; LF*, 5′-labeled with biotin used in LAMP-LFB assay

### The standard LAMP-LFB assay

The LAMP-LFB reaction was performed in the standard LAMP conditions, which have been described previously (Gandelman et al. 2011; Wang et al. 2014). This assay was used to determine the suitability of the *M. kansasii*-LAMP primers. The LAMP reaction (25 μL amplification system) contained the following components: 1.4 μL primers comprised 0.1 μM F3, 0.1 μM B3, 0.4 μM FIP*, 0.4 μM BIP, 0.2 μM LF*, 0.2 μM LB, 12.5 μL 2× reaction mix buffer mix from the Isothermal Amplification Kits (HaiTaiZhengYuan Technology Co., Ltd., Beijing, China), 1.0 μL *Bst* DNA polymerase (10 U) from the Isothermal Amplification Kits, 1.0 μL DNA template, and 9.1 μL ddH_2_O (1.0 μL of colorimetric indicator and 8.1 μL ddH_2_O when using colorimetric indicator method). The reaction products were confirmed using the following methods: colorimetric indicator analysis, turbidimeters (LA-320C), and LFB detection.

When using colorimetric indicator method, we evaluated the results and expected the color of the amplified products to change from violet to azure in the positive tube, while negative controls and blank controls remained violet. When using LAMP-LFB assay, the positive responses should indicate two visible red lines (test line, TL; control line, CL), while negative and blank controls showed only the control lines.

In these experiments, *M. kansasii* (ATCC12478) was used as the positive strain, *M. tuberculosis* H37Rv (ATCC 27294) was the negative control, and double distilled water was the blank control. To verify the optimal amplification temperature, at which there was the fastest reaction rate, a total of six temperatures (65–70 °C, with 1 °C interval) were compared under standard LAMP protocol for 60 min, then hold the reaction at 85 °C for 5 min. The reaction products were analyzed by LFB detection.

### Specificity and detection limit of the *M. kansasii*-LAMP-LFB assay

The specificity of the *M. kansasii*-LAMP-LFB was analyzed using the DNA template from 25 *M. kansasii* strains, 60 mycobacterial strains, and 3 strains from other respiratory bacteria (Table 1). These tests were repeated three times. The detection limit was determined using serial dilutions (1 ng, 10 pg, 1 pg, 100 fg, 10 fg, 1 fg, and 0.1 fg per microliter) of the genomic DNA template. The results of the *M. kansasii*-LAMP-LFB were compared with values collected using the colorimetric indicator and real-time turbidity methods. Three independent replicates were tested.

### Optimal amplification time for the *M. kansasii*-LAMP-LFB assay

The optimal amplification time for the *M. kansasii*-LAMP-LFB was confirmed by increasing the reaction time from 30 to 40 min, at 10-min intervals, and then comparing those results with that obtained from the experiment with a 60-min reaction time. The reaction products were then analyzed by LFB detection. Three independent replicates were analyzed.

### *M. kansasii*-LAMP-LFB assay testing artificial sputum samples

The above experiments were used to determine optimum amplification temperature and time. Based on these optimal reaction conditions of the *M. kansasii*-LAMP-LFB assay, artificial sputum samples of *M. kansasii* (ATCC 12478) and *M. tuberculosis* H37Rv (ATCC 27294) were used to evaluate its accuracy.

The artificial sputum used in this study was obtained from the National Tuberculosis Reference Laboratory of China (NTRL). Its main components were normal saline, sterile purified water, lecithin, salmon sperm DNA, bovine serum albumin, and mucin (precise composition was not listed because of the patents). *Mycobacterium kansasii* solution (5 μL), at a concentration of 10^5^ CFU/mL, was added into the tube with 995 μL artificial sputum medium, and at last, the concentration of *M. kansasii* artificial sputum sample was 500 CFU/mL. The *M. kansasii* DNA was extracted from *M. kansasii* artificial sputum sample by the boiling method using 100 μL ddH_2_O. And then, 5 μL of DNA template solution from the 100 μL boiling method product was used in the 25 μL amplification system. There were only 25 CFU *M. kansasii* DNA in the amplification system at the end. According to above method, 10 μL 10^5^ CFU/mL of *M. kansasii* added to 990 μL artificial sputum medium to obtain 1000 CFU/mL *M. kansasii* artificial sputum sample, and 5 μL DNA template solution in the final 25 μL amplification system comprised just 50 CFU bacteria DNA. Then using the above method, 5 μL template with 5000 CFU and 500 CFU *M. kansasii* strain DNA was obtained.

Finally, in the assay itself, 5 μL DNA template derived from *M. kansasii* strains (5000 CFU, 500 CFU, 50 CFU, 25 CFU) and a *M. tuberculosis* H37Rv strain (5000 CFU) were used in the 25 μL amplification system to evaluate the assay.

## Results

### Optimization of the temperature for *M. kansasii*-LAMP-LFB assay

*Mycobacterium kansasii* ATCC12478 DNA (1 ng per reaction) was used as the positive control, to verify the optimal amplification temperature. When the assay was carried out at temperatures ranging from 65°C to 70 °C, the reaction was monitored by the real-time turbidity. This experiment demonstrated that the most suitable temperature was 67 °C, at which the shortest reaction time was observed (Figure S2).

### Detection limit and optimal amplification time for the *M. kansasii*-LAMP-LFB assay

**Fig. 1:**
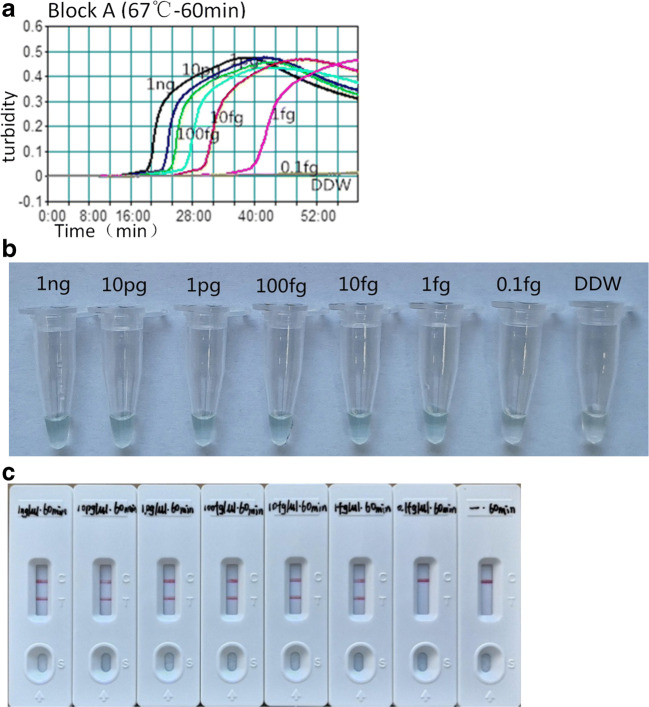
The detection limit of the *M. kansasii*-LAMP assay. Serial dilutions (1 ng/μL, 10 pg/μL, 1 pg/μL, 100 fg/μL, 10 fg/μL, 1 fg/μL, 0.1 fg/μL) of the *M. kansasii* DNA template were used, and amplification products were monitored by turbidimeters (**a**), colorimetric indicator (**b**), and LFB detection (**c**)

Serial-diluted (1 ng/μL, 10 pg/μL, 1 pg/μL, 100 fg/μL, 10 fg/μL, 1 fg/μL, 0.1 fg/μL) *M. kansasii* genomic DNA samples were used in the *M. kansasii*-LAMP assay at 67 °C. According to the results, our method could successfully amplify and detect the DNA samples at concentrations as low as 1 fg/mL (Fig. 1). The optimal amplification time was 40 min by LFB detection (Figure S3).

### Specificity of *M. kansasii*-LAMP-LFB assay

**Fig. 2:**
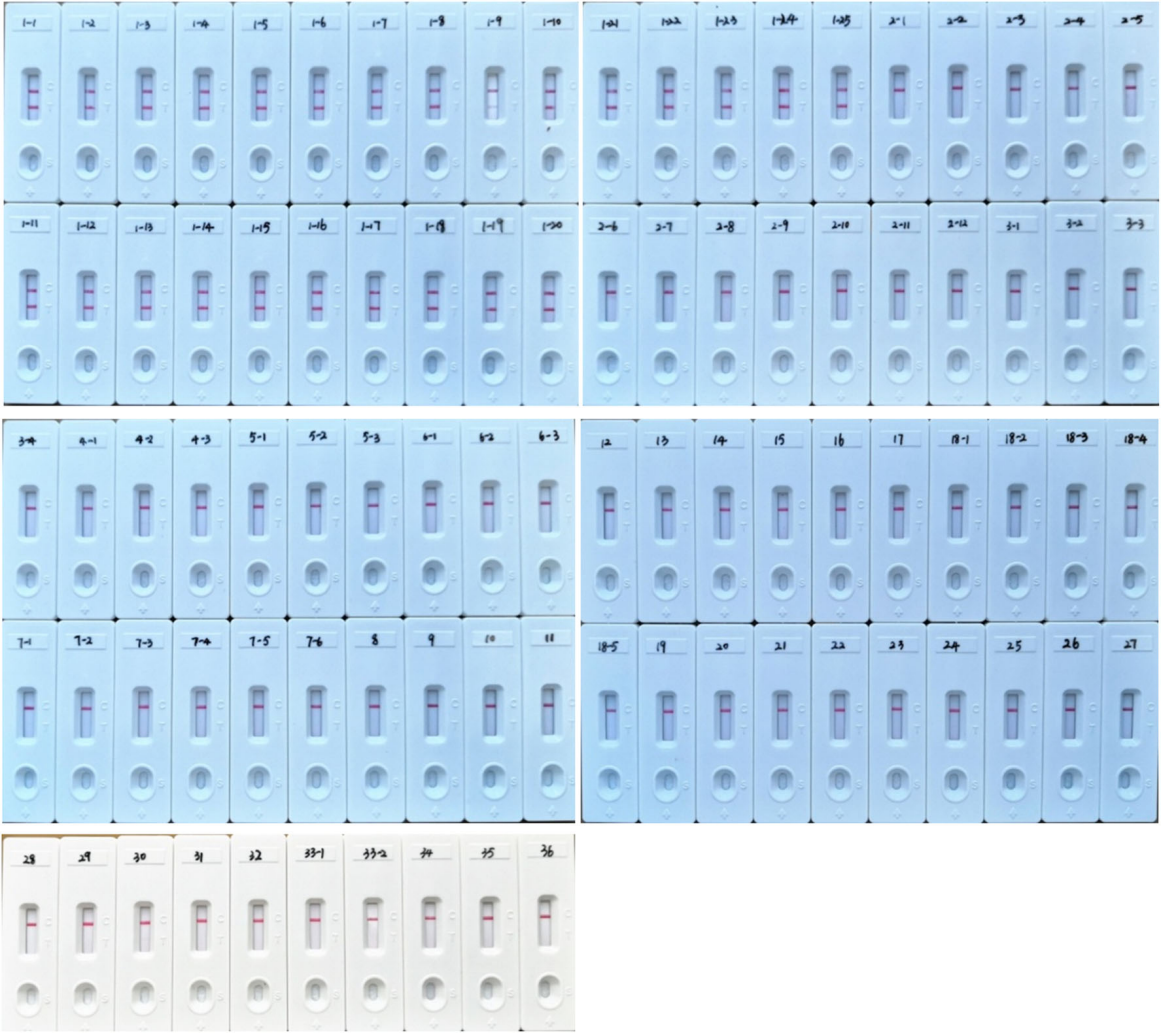
The specificity of the *M. kansasii*-LAMP-LFB assay. Biosensor 1-1, *M. kansasii* (ATCC12478); biosensor 1-2 ~ 1-25, *M. kansasii* isolated strains (NTRL); biosensor 2-1, *M. tuberculosis* H37Rv (ATCC27294); biosensors 2-2 ~ 2-12 *M. tuberculosis* isolated strains (NTRL); biosensor 3-1, *M. intracellulare* (ATCC13950); biosensors 3-2 ~ 3-4, *M. intracellulare* isolated strains (NTRL); biosensor 4-1, *Mycobacterium chelonae* (ATCC14472); biosensors 4-2 ~ 4-3, *M. chelona*e isolated strains (NTRL); biosensor 5-1, *Mycobacterium fortuitum* (ATCC6841); biosensors 5-2 ~ 5-3, *M. fortuitum* isolated strains (NTRL); biosensor 6-1, *Mycobacterium gordonae* (ATCC14470); biosensors 6-2 ~ 6-3, *M. gordonae* isolated strains (NTRL); biosensor 7-1, *M. abscessus* (ATCC19977); biosensors 7-2 ~ 7-6, *M. abscessus* isolated strains (NTRL); biosensor 8, *Mycobacterium aurum* (ATCC23366); biosensor9, *Mycobacterium neoaurum* (ATCC25795); biosensor 10, *Mycobacterium marinum* (ATCC927); biosensor 11, *Mycobacterium gilvum* (ATCC43909); biosensor 12, *Mycobacterium aichiense* (ATCC27280); biosensor 13, *Mycobacterium smegmatis* (ATCC19420); biosensor 14, *Mycobacterium parafortuitum* (ATCC19686); biosensor 15, *Mycobacterium terrae* (ATCC15755); biosensor 16, *Mycobacterium nonchromogenicum* (ATCC19530); biosensor 17, *Mycobacterium vaccae* (ATCC15483); biosensor 18-1, *Mycobacterium avium* (ATCC252910; biosensors 18-2 ~ 18 ~ 5 isolated strains (NTRL); biosensor 19, *Mycobacterium phlei* (ATCC11758); biosensor 20, *Mycobacterium scrofulaceum* (ATCC19981); biosensor 21, *Mycobacterium gastri* (ATCC15754); biosensor 22, *Mycobacterium triviale* (ATCC23292); biosensor 23, *Mycobacterium xenopi* (ATCC19250); biosensor 24, *Mycobacterium africanum* (ATCC25420); biosensor 25, *Mycobacterium bovis* BCG (ATCC 19274); biosensor 26, *M. tuberculosis* H37Ra (ATCC 25177); biosensor 27, *M. bovis* (ATCC19210); biosensor 28, *Mycobacterium malmoense* (ATCC29571); biosensor 29, *Mycobacterium arupense* isolated strains (NTRL); biosensor 30, *Mycobacterium kumamotonense* isolated strains (NTRL); biosensor 31, *Mycobacterium paragordonae* isolated strains (NTRL); biosensor 32, *M. scrofulaceum*isolated strains (NTRL); biosensors 33-1 ~ 33-2, double distilled water; biosensor 34, *K. pneumoniae* isolated strains (NTRL); biosensor 35, *N. meningitidis* isolated strains (NTRL); biosensor 36, *S. pneumonia* isolated strains (NTRL). National Tuberculosis Reference Laboratory of CHINA, NTRL

A total of 1 ng of all strains in Table 1 was used in the *M. kansasii*-LAMP-LFB assays at 67 °C for 40 min. The DNA from *M. kansasii* ATCC 12478 and 24 clinical isolated strains had positive results. The other mycobacteria species, as well as the three respiratory pathogenic bacteria (*Klebsiella pneumoniae*, *Neisseria meningitidis*, *Streptococcus pneumoniae*), and double distilled water were negative (Fig. 2). On the lateral flow biosensor, two red lines (TL and CL) appeared positive, and only one red line (CL) appeared negative. The above results suggest the assay could specifically detect and focus on *M. kansasii* relative to other mycobacteria and other common respiratory pathogens.

### Sensitivity of *M. kansasii*-LAMP-LFB assay of artificial sputum samples

**Fig. 3:**
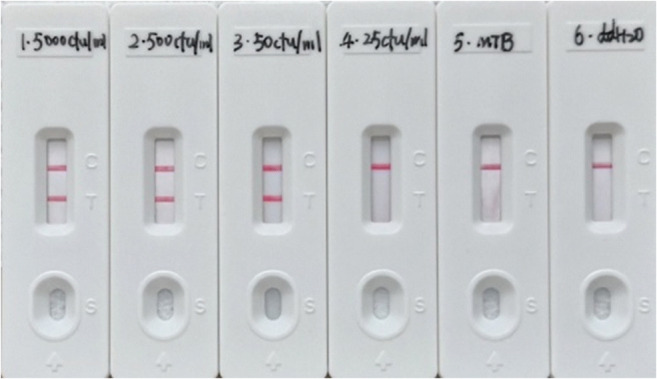
The sensitivity of *M. kansasii*-LAMP-LFB assay of sputum samples. 1, 5000 CFU *M. kansasii* strain DNA in the assay; 2, 500 CFU *M. kansasii* strain DNA in the assay; 3, 50 CFU *M. kansasii* strain DNA in the assay; 4, 25 CFU *M. kansasii* strain DNA in the assay; 5, 5000 CFU *M. tuberculosis* strain DNA in the assay; 6, blank control with 5 μL double distilled water

DNA template (5 μL) was added in the *M. kansasii*-LAMP-LFB assay, and 50 CFU of the specific *M. kansasii* strain could be detected in the sputum by the lateral flow biosensor (Fig. 3).

## Discussion

*Mycobacterium kansasii* infection was first detected in Kansas City, USA in 1953. *Mycobacterium kansasii* is a photochromogenic bacterium and is one of the most important nontuberculous mycobacteria found in clinical settings (Zofia et al. 2018). It is pathogenic to humans, and primarily causes human pulmonary infections and extrapulmonary disseminated infections (James et al. 2017; Kaur et al. 2011; Moon et al. 2015). The isolation rate of *M. kansasii* is very high in Europe, America, and Japan, especially in patients with acute immunodeficiency syndrome (Basille et al. 2018; Furuuchi et al. 2019; Gary 2017; Santin et al. 2018). In a report, in distribution of nontuberculous mycobacteria species isolated from pulmonary NTM patients in South China, the separation rate of *M. kansasii* was ranked forth, following *Mycobacterium abscessus*, *Mycobacterium intracellulare*, and *Mycobacterium avium* (Tan et al. 2018). It is therefore necessary to detect *M. kansasii* using rapid and accurate detection methods. The method that we developed in this study is a simple, rapid, specific, and inexpensive constant temperature amplification method. Constant temperature gene amplification can save time during the amplification and detection steps (Gandelman et al. 2011; Tomita et al. 2008).

In this study, we chose a specific gene, *rpoB* of *M. kansasii*, to design the LAMP primers. Using BLAST analysis, we found that the *rpoB* sequence we selected is highly species specific for detecting *M. kansasii*. The *M. kansasii*-LAMP-LFB assay showed positive results with only *M. kansasii*, suggesting that this assay is highly specific. In the assay, the amplification step was conducted at 67 °C for 40 min and stopped while held at 85°C for 5 min. The amplified products were added to the LFB and the results could be seen in almost 2 min. The whole detection process from isothermal amplification to result observation only needs about 50 min, which is significantly faster than other molecular methods like GeneChip, reverse hybridization, and sequencing technology. Moreover, it showed excellent sensitivity and a detection limit of 1 fg, allowing even 50 CFU of *M. kansasii* strains in sputum to be tested. For the entirety of this experimental process, only a constant temperature machine was needed. The requirements for the equipment were extremely simple, and it was very convenient to observe the results without any additional apparatus required. Notably, the whole process cost including primers, isothermal amplification reagents, and lateral flow biosensor is only about 10 dollars. Therefore, the *M. kansasii*-LAMP-LFB assay could be a very simple, fast, specific, and inexpensive way to detect clinical disease caused by *M. kansasii* infection. And also, this is the first time LAMP-LFB used for testing *M. kansasii*.

## Electronic supplementary material

ESM 1 (DOCX 617 kb)

## Data Availability

All data used or analyzed during this study are included within this article.
