# Supplementary material for: Detection of Mycobacterium kansasii using a combination of loop-mediated isothermal amplification (LAMP) and lateral flow biosensors
Source: Int Microbiol. 2020 Sep 2;24(1):75–82. doi: 10.1007/s10123-020-00143-z (PMC7872997; doi:10.1007/s10123-020-00143-z)

Supplementary Materials

**Detection of *Mycobacterium kansasii* Using a Combination of Loop-Mediated Isothermal Amplification (LAMP) and Lateral Flow Biosensors**

Chuang Chen^1^, Jia Lu^1^, Bo Long^1^, Zhengyuan Rao^1^, Yuan Gao^1^, Weina Wang^1^, Wenfeng Gao^1^, Jun Yang^1^, Shu Zhang^1*^

^1^Sichuan Center for Disease Control and Prevention, Chengdu, Sichuan Province, China

*Corresponding author:

Shu Zhang, Sichuan Center for Disease Control and Prevention, Chengdu, 610041, Sichuan Province, China

Tel/fax: +86 028 85586535

E-mail: dulianyouchao@qq.com

**Content**

**Figure S1.** Sequence of *rpoB* gene (*M. kansasii* ATCC12478) and location of primers used for the LAMP-LFB assay.

**Figure S2**.Optimal amplification temperature for the *M. kansasii*-LAMP assay. Each reaction was monitored by real-time turbidity method.

**Figure S3**.Optimal amplification time for the *M. kansasii*-LAMP assay.

**Figure S1.** Sequence of *rpoB* gene (*M. kansasii* ATCC12478) and location of primers used for the LAMP-LFB assay.


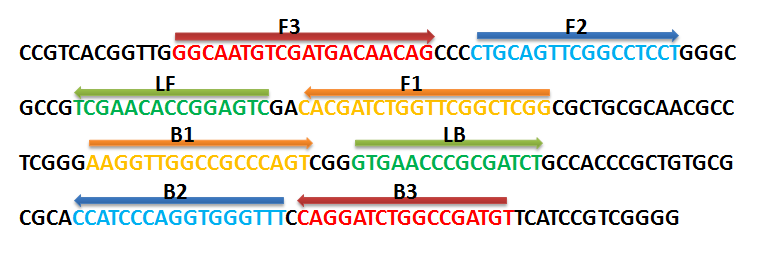


Note: Arrows pointing to the right arrows indicate the sense strand; arrows pointing to the left indicate the complementary sequences.

**Figure S2**.Optimal amplification temperature for the *M. kansasii*-LAMP assay. Each reaction was monitored by real-time turbidity method.


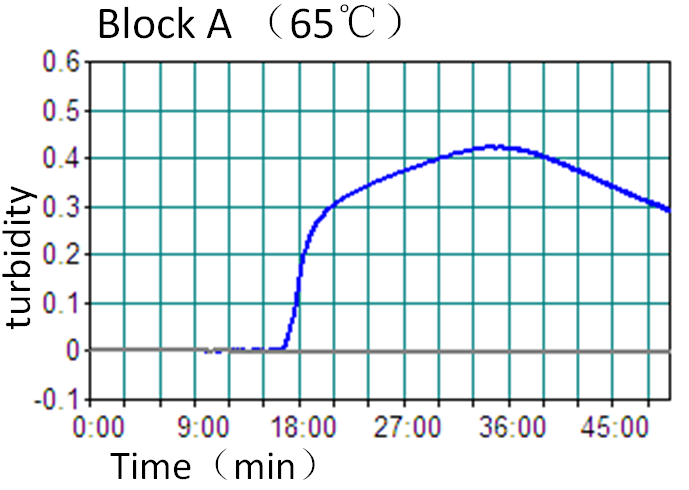

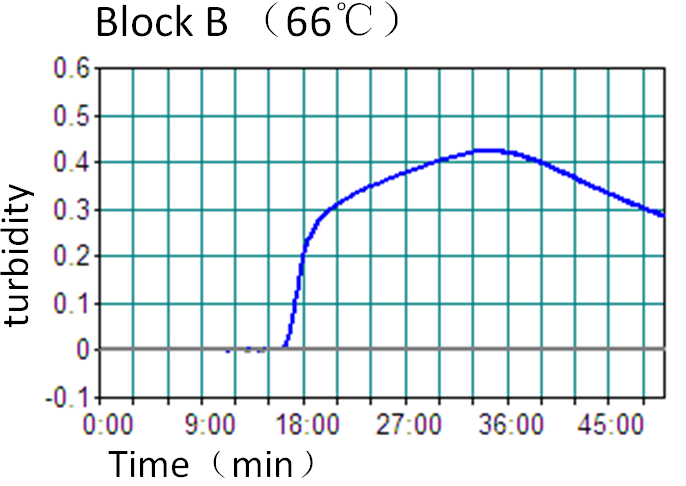


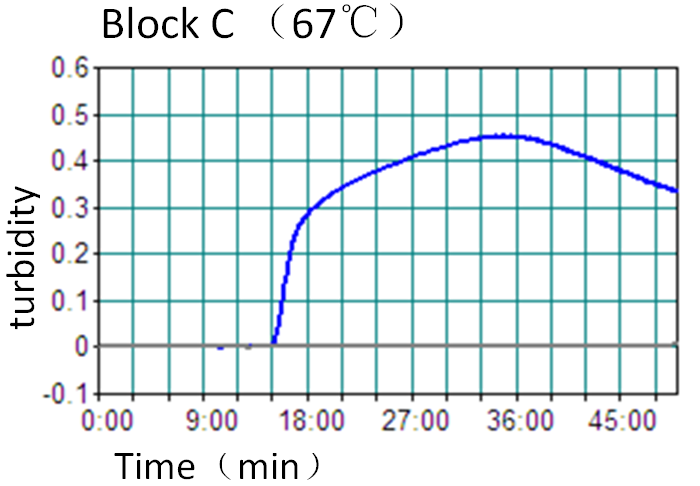

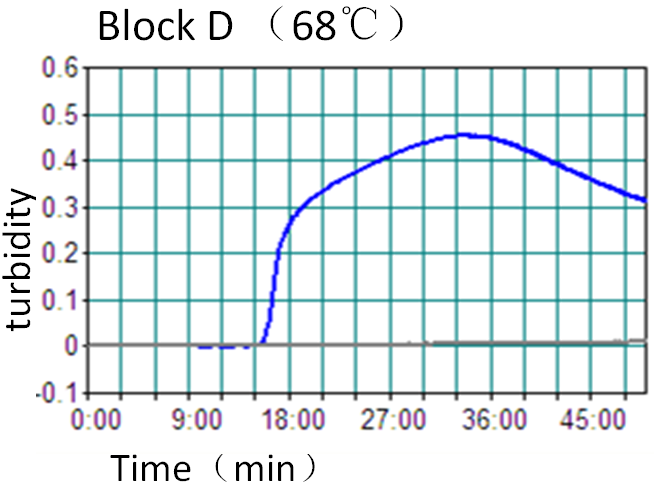


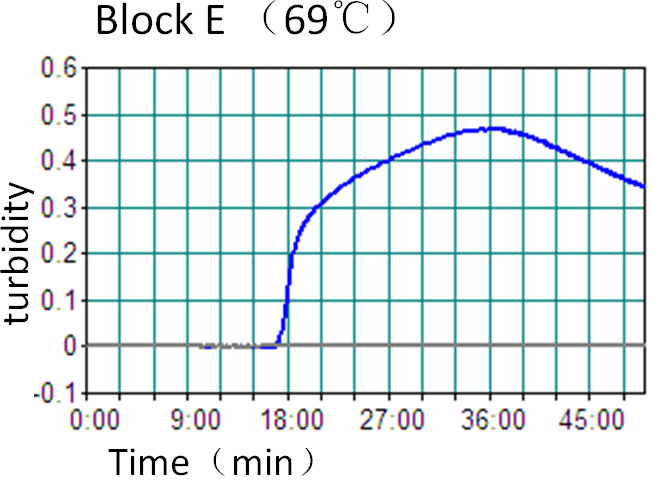

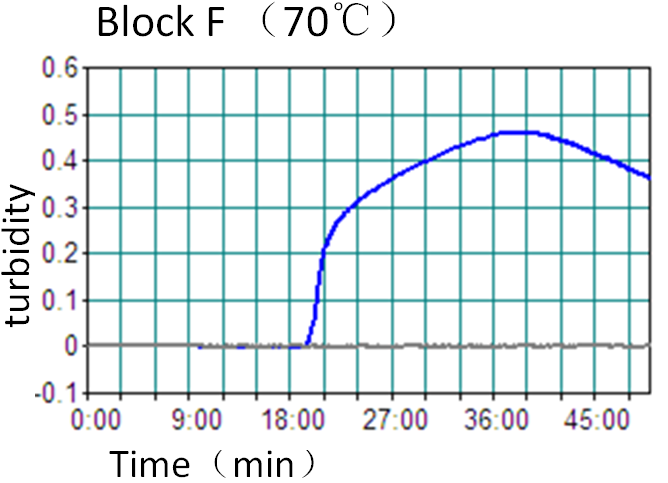


**Figure S3**. Optimal amplification time for the *M. kansasii*-LAMP assay.

Serial dilutions (1 ng/μL, 10 pg/μL, 1 pg/μL,100 fg/μL, 10 fg/μL, 1 fg/μL, 0.1 fg/μL) of the *M. kansasii* DNA template were used, and the products were monitored by LFB detection.


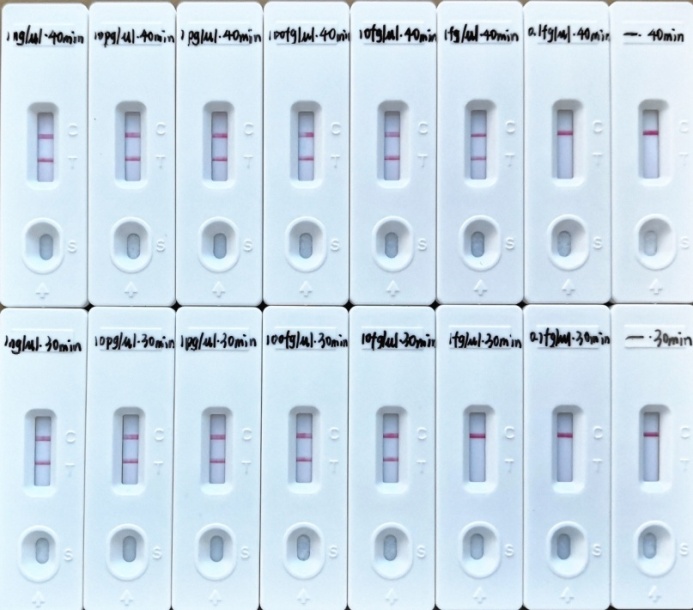

Supplement: Supplementary file 1 — (DOCX 617 kb) [file 10123_2020_143_MOESM1_ESM.docx]
